# Risks of ventilator-associated pneumonia and invasive pulmonary aspergillosis in patients with viral acute respiratory distress syndrome related or not to Coronavirus 19 disease

**DOI:** 10.1186/s13054-020-03417-0

**Published:** 2020-12-18

**Authors:** Keyvan Razazi, Romain Arrestier, Anne Fleur Haudebourg, Brice Benelli, Guillaume Carteaux, Jean‑Winoc Decousser, Slim Fourati, Paul Louis Woerther, Frederic Schlemmer, Anais Charles-Nelson, Francoise Botterel, Nicolas de Prost, Armand Mekontso Dessap

**Affiliations:** 1 AP-HP (Assistance Publique-Hôpitaux de Paris), Hôpitaux universitaires Henri Mondor, DMU Médecine, Service de Médecine Intensive Réanimation, 94010 Créteil, France; 2 UPEC (Université Paris Est Créteil), Faculté de Santé de Créteil, IMRB, GRC CARMAS, 94010 Créteil, France; 3 UPEC (Université Paris Est), INSERM, Unité U955, 94010 Créteil, France; 4 AP-HP (Assistance Publique-Hôpitaux de Paris), Hôpitaux universitaires Henri Mondor, Contrôle, Epidémiologie et Prévention de l’Infection, CEPI, 94010 Créteil, France; 5 AP-HP (Assistance Publique-Hôpitaux de Paris), Hôpitaux universitaires Henri Mondor, Département de Virologie, Bactériologie, Parasitologie-Mycologie, 94010 Créteil, France; 6 UPEC (Université Paris Est), EA 7380 Dynamic, Ecole nationale vétérinaire d’Alfort, USC Anses, Créteil, France; 7 AP-HP (Assistance Publique-Hôpitaux de Paris), Hôpitaux universitaires Henri Mondor, DHU A-TVB, Unité de Pneumologie, 94010 Créteil, France; 8 AP-HP (Assistance Publique-Hôpitaux de Paris), Hôpital européen Georges Pompidou, Unité d’Épidémiologie et de Recherche Clinique, INSERM, Centre d’Investigation Clinique1418, module Épidémiologie Clinique, Paris, France

**Keywords:** COVID-19, ARDS, Nosocomial pneumonia, Ventilator-associated pneumonia, Invasive aspergillosis

## Abstract

**Background:**

Data on incidence of ventilator-associated pneumonia (VAP) and invasive pulmonary aspergillosis in patients with severe SARS-CoV-2 infection are limited.

**Methods:**

We conducted a monocenter retrospective study comparing the incidence of VAP and invasive aspergillosis between patients with COVID-19-related acute respiratory distress syndrome (C-ARDS) and those with non-SARS-CoV-2 viral ARDS (NC-ARDS).

**Results:**

We assessed 90 C-ARDS and 82 NC-ARDS patients, who were mechanically ventilated for more than 48 h. At ICU admission, there were significantly fewer bacterial coinfections documented in C-ARDS than in NC-ARDS: 14 (16%) vs 38 (48%), *p* < 0.01. Conversely, significantly more patients developed at least one VAP episode in C-ARDS as compared with NC-ARDS: 58 (64%) vs. 36 (44%), *p* = 0.007. The probability of VAP was significantly higher in C-ARDS after adjusting on death and ventilator weaning [sub-hazard ratio = 1.72 (1.14–2.52), *p* < 0.01]. The incidence of multi-drug-resistant bacteria (MDR)-related VAP was significantly higher in C-ARDS than in NC-ARDS: 21 (23%) vs. 9 (11%), *p* = 0.03. Carbapenem was more used in C-ARDS than in NC-ARDS: 48 (53%), vs 21 (26%), *p* < 0.01. According to AspICU algorithm, there were fewer cases of putative aspergillosis in C-ARDS than in NC-ARDS [2 (2%) vs. 12 (15%), *p* = 0.003], but there was no difference in *Aspergillus* colonization.

**Conclusions:**

In our experience, we evidenced a higher incidence of VAP and MDR-VAP in C-ARDS than in NC-ARDS and a lower risk for invasive aspergillosis in the former group.

## Background

The pandemic of severe acute respiratory coronavirus 2 (SARS-CoV-2), responsible of coronavirus disease 2019 (COVID-19), has resulted in high rates of hospitalization and intensive care unit (ICU) admission to treat acute respiratory distress syndrome (ARDS).

There is scarce information in the literature on the rates of coinfections in COVID-19 patients. A total of 62/806 (8%) cases of bacterial/fungal coinfection were reported in nine cohort studies on COVID-19 patients [1]. These studies did not uniformly report bacterial coinfection, thus potentially underestimate the rates of respiratory coinfections. Broad-spectrum antibacterial therapy was widely used in more than 90% of COVID-19 cases receiving antibacterial therapy in ICU [2, 3]. However, there has been no robust report on ventilator-associated pneumonia (VAP) in COVID-19-associated ARDS patients (C-ARDS) to date. ARDS is a known risk factor for VAP but little is known about C-ARDS in this concern. Similar to severe influenza complications, recent reports have documented invasive pulmonary aspergillosis in COVID-19 patients but given no real incidence analysis [4, 5].

We conducted a retrospective study aimed at comparing the incidence of VAP and of invasive pulmonary aspergillosis between patients with C-ARDS and those with non-SARS-CoV-2 viral ARDS (NC-ARDS).

## Methods

### Setting and patients

We conducted a cohort study which retrospectively enrolled, between October 1, 2009, and April 29, 2020, all patients referred to the medical ICU of a French tertiary hospital for viral ARDS and who required mechanical ventilation for more than 48 h. ARDS was defined according to the Berlin definition [6]. C-ARDS were patients with ARDS and a positive polymerase chain reaction (PCR) test for SARS-COV2. NC-ARDS patients were those having ARDS and a positive polymerase chain reaction (PCR) test for influenza, human metapneumovirus, respiratory syncytial virus, parainfluenza virus, other endemic human coronaviruses (OC43, NL63, HK-U1, 229E), and adenovirus. RT-PCR duplex targeting influenza A/B, respiratory syncytial virus, metapneumovirus, coronavirus, adenovirus, and parainfluenza were performed using r-gene™ (Argene, Biomérieux S.A.) according to the manufacturer’s instructions. We carefully identified all patients with viral ARDS using a triple check involving the ICU medical reports, the medical information system database, and the virology department registry. Of note, respiratory viruses were systematically searched in all patients admitted in our ICU for pneumonia. This observational study was approved by the Institutional Review Board of the French intensive care medicine society (CE SRLF 20-45) and informed consent was waived.

The mechanical ventilation of ARDS patients followed a standardized protective ventilation strategy [7]. Other treatments, including neuromuscular blocking agents, inhaled nitric oxide, prone positioning, and veno-venous extra-corporeal membrane oxygenation were administered with respect to national guidelines [8]. Sedation protocol was based on the adaptation of the dose of sedative (midazolam or propofol) on the Richmond Agitation Sedation Scale by the nurse every three hours and did not significantly changed during the study period. Preventing VAP followed an educational program, regular reminders and feedback with a prevention care bundle in accordance with guidelines [9], based on hand hygiene with alcohol-based sanitizer, inclining patients in a semi-recumbent position (30°–45°), oral chlorhexidine mouth washing at least four times a day, tracheal cuff pressure maintenance between 20 and 30 cm H_2_O, orogastric rather than nasogastric tubes, and daily chlorhexidine body washing. No routine antibiotic prophylaxis or decontamination antibiotic regimens were prescribed and if stress ulcer prophylaxis was needed, proton pump inhibitor was preferred. All patients had a closed tracheal suction system without daily change.

### Demographic, clinical, and laboratory data

Demographic characteristics, comorbidities, Charlson comorbidity index [10], clinical, biological, and imaging features at ICU admission, and consumption of alcohol-based handrub liquid (retrieved from hospital pharmacy) was collected, then analyzed on May 25, 2020, after a minimal follow-up period of 28 days for the most recent patients. Respiratory tract secretions were cultured for VAP diagnosis purposes, and susceptibility profiles of recovered microorganisms were recorded.

The primary endpoint was the difference in incidence of first VAP between C-ARDS and NC-ARDS patients. VAP was clinically suspected if any of its classical criteria happened 48 h or more after mechanical ventilation initiation: new or worsening infiltrates on chest roentgenogram, systemic signs of infection (new-onset fever, leukocytosis or leucopenia, increased need for vasopressors to maintain blood pressure), purulent secretions, and impaired oxygenation [11]. All suspected VAP were confirmed from quantitative cultures of lower respiratory tract secretions sampled before administering new antibiotics using a blinded protected telescope catheter [12] or bronchoscopy (10^3^ and 10^4^ colony forming units/mL for protected telescope catheter and bronchoalveolar lavage, respectively). VAP onset was defined as the day on which the lung sample tested positive. The secondary endpoints were the difference in occurrence of bacterial coinfection at ICU admission, putative invasive pulmonary aspergillosis, and multi-drug-resistant bacteria (MDR) VAP. MDR pathogens included methicillin-resistant *Staphylococcus aureus*, extended-spectrum β-lactamase-producing *Enterobacteriaceae* (ESBL-PE), and carbapenem-resistant *Enterobacteriaceae* (CRE). During the study period, our management of VAP/hospital-acquired pneumonia (HAP) was derived from that recommended by the French guidelines to treat adult ICU-acquired pneumonia [13]. Bacterial coinfection at ICU admission was evidenced by the detection of bacteria in the sputum or in blood samples, in the absence of other sources of infection, or by a positive pneumococcal or *L. pneumophila* serotype 1 urinary antigen test.

We based our definition of invasive pulmonary aspergillosis on one of the following: (1) the recently published influenza‑associated pulmonary aspergillosis definition by expert panel (Influenza-Associated Pulmonary Aspergillosis—IAPA case definition) [14]; (2) the crude AspICU algorithm, as proposed by Blot et al. to distinguish putative invasive pulmonary aspergillosis from respiratory tract *Aspergillus spp.* colonization in critically-ill patients, relying on clinical, radiological, and mycological criteria [15]; (3) the modified *AspICU* algorithm of invasive pulmonary aspergillosis, which includes the positivity of serum or bronchoalveolar lavage galactomannan, as proposed by the Dutch-Belgian Mycosis study group to define invasive pulmonary aspergillosis in critically ill patients with severe influenza [16].

### Statistical analysis

No statistical sample size calculation was performed a priori, and sample size was equal to the number of patients treated during the study period. The results are reported as median and interquartile range (25th–75th percentiles) or numbers with percentages. Initial bivariate statistical comparisons were conducted using *χ*^2^ or Fisher’s exact tests for categorical data and Mann–Whitney *U* test for continuous data.

To identify risk factors for VAP and invasive aspergillosis, we used classical multivariable logistic regression with a backward procedure, because the role of competing events like extubation and death was not relevant for this analysis. Non-redundant variables selected in bivariate analysis (*p* < 0.10) and considered clinically relevant were entered into the logistic regression model. Variables included in the final model for VAP were male gender, C-ARDS, congestive heart failure (NYHA 3-4), SAPS II at ICU admission, and bacterial coinfection at ICU admission. The results are expressed as crude and adjusted odd ratios (OR) with their 95% confidence intervals (CI).

### Competing risks analysis

As the risk of VAP cumulatively increases over time of mechanical ventilation, death and ventilator weaning are competing risks for VAP occurrence [17]. Patients are no longer at risk for VAP after death or ventilator weaning; conversely, weaning may be prolonged because of VAP. In this context, standard survival methods (Kaplan–Meier method and Cox model) are inappropriate because they assume that censoring is non-informative [18], hence the need to consider specific competing risk methods. We therefore used a competing risk model (cumulative incidence function of the Gray model) [19, 20] to properly estimate the effect of COVID-19 on VAP development, while considering death and ventilator weaning as competing events. The strength of the association between each variable and the outcome was assessed with the sub-hazard ratio and the cumulative incidence function, estimated using cmprsk package developed by Gray in R software (http://biowww.dfci.harvard.edu/~gray/cmprsk_2.1-4.tar.gz). Because there were differences between the two groups, resulting from the particular profile of patients prone to have severe forms of COVID-19 (e.g., cardiovascular comorbidities) or non-COVID-19 viral pneumonia (e.g., immunosuppression), we further matched C-ARDS and NC-ARDS and performed a sensitivity analysis as follows. First, we screened all differences between groups. Second, we used a seminal review summarizing risk factors for VAP [21], to select among variables that were different between the two groups, those that could explain more VAP in the C-ARDS group as compared to the NC-ARDS group. Following this process, 68 C-ARDS patients could be matched 1:1 to 68 NC-ARDS for ARDS severity (mild, moderate, or severe) and diabetes mellitus. Of note, these matched pairs were comparable regarding age and gender. A sensitivity analysis with competing risk was performed in this matched cohort. Two-sided *p* values < 0.05 were considered significant. The other analyses were conducted using SPSS Base 21.0 statistics software package (SPSS Inc., Chicago, IL).

## Results

### The study

Between October 1, 2009, and April 29, 2020, 3821 consecutive patients were mechanically ventilated in our ICU. Among them, 199 had a viral positive PCR, including 172 pneumonia with ARDS criteria that were mechanically ventilated for more than 48 h. Ninety patients had C-ARDS with positive real-time reverse transcriptase PCR tests for COVID-19, while 82 patients had NC-ARDS, including 50 with severe influenza (with two respiratory syncytial virus coinfections), six with endemic human coronavirus (with one respiratory syncytial virus coinfection), 14 with respiratory syncytial virus alone, five with human metapneumovirus, five with parainfluenza, and two with adenovirus. Thus, the present study comprises 90 patients with C-ARDS ad 82 with NC-ARDS (Additional file 1: Figure S1).

### Patients’ characteristics

The characteristics of C-ARDS and NC-ARDS patients are displayed in Table 1. NC-ARDS patients had worse past history (Mc Cabe classification and Charlson comorbidity index) and were often immunosuppressed, whereas C-ARDS patients were often diabetic and hypertensive males. At ICU admission almost all patients received antibiotics in both groups, but C-ARDS received less steroid had less organ failure (as assessed by Sequential Organ Failure Assessment score), and lower PaO_2_/FiO_2_ ratio than NC-ARDS patients. During ICU stay, C-ARDS patients more often required neuromuscular blockade, prone positioning, nitric oxide inhalation, extra-corporeal membrane oxygenation support, and longer duration of mechanical ventilation than NC-ARDS patients. In-ICU and day 28 mortalities were similar in both.

**Table 1:** Characteristics of patients with acute respiratory distress syndrome related to Coronavirus disease 19 (C-ARDS) or other viruses (NC-ARDS)

Variables	NC-ARDS (*n* = 82)	C-ARDS (*n* = 90)	*p* value
Age, median [IQR]	63 [57–71]	59 [53–69]	0.09
Male gender	54 (66%)	74 (82%)	0.01
**Medical history**			
Mc Cabe and Jackson classification			< 0.001
No underlying disease	47 (57%)	76 (84%)	
Ultimately fatal	24 (29%)	12 (13%)	
Rapidly fatal disease	11 (14%)	2 (2%)	
Charlson comorbidity index	2 [1–3]	1 [0–2]	< 0.001
Diabetes mellitus	23 (28%)	39 (43%)	0.037
Congestive heart failure (NYHA 3–4)	6 (7%)	7 (8%)	0.91
Supraventricular arrhythmia	12 (15%)	8 (9%)	0.24
Hypertension	36 (44%)	59 (66%)	0.004
COPD	10 (12%)	9 (10%)	0.64
Chronic renal failure	11 (13%)	14 (16%)	0.69
Dialysis	3 (4%)	2 (2%)	0.67
Stroke	5 (6%)	4 (4%)	0.74
Liver cirrhosis (Child C)	1 (1%)	0	0.48
Current smoking	22 (27%)	25 (28%)	0.89
**Immunosuppression conditions**	40 (49%)	16 (18%)	< 0.001
Solid cancer	4 (5%)	5 (6%)	0.99
Blood cancer	17 (21%)	1 (1%)	< 0.001
Organ transplant	9 (11%)	5 (6%)	0.19
HIV infection	4 (5%)	3 (3%)	0.71
Sickle cell disease	2 (2%)	3 (3%)	0.99
Others	5 (6%)	2 (2%)	0.26
***Clinical characteristics upon ICU admission***			
SAPS II	49 [37–67]	36 [27–45]	< 0.001
Baseline SOFA—median [IQR]	9 [5–12]	7 [4–8]	< 0.001
PaO_2_/FiO_2_ ratio (mmHg) median [IQR]	162 [101–210]	120 [92–163]	0.005
ARDS classification (Berlin definition)			0.018
Mild	24 (29%)	11 (12%)	
Moderate	39 (48%)	49 (54%)	
Severe	19 (23%)	30 (33%)	
Norepinephrine, *n* (%)	43 (52%)	42 (47%)	0.45
Serum creatinine (µmol/L)	108 [72–195]	83 [6–128]	0.004
White blood cell count (× 10^9^/L)	7.5 [0–15]	8.2 [5–12]	0.49
Lymphocyte count (× 10^9^/L)	0.6 [0.3–1.1]	0.8 [0.5–1.2]	0.03
Lymphocyte count (× 10^9^/L) in non-immunocompromised patients	0.8 [0.4–1.2]	0.8 [0.5–1.2]	0.62
Documented bacterial coinfection	38 (48%)	14 (16%)	< 0.001
**Treatment during the first 24 h**			
Antibiotics	81 (99%)	90 (100%)	0.48
Antiviral treatment	58 (71%)	69 (76%)	0.39
Corticosteroids (any dose)*	30/81 (37%)	12/87 (14%)	0.001
Corticosteroids (low dose)*^#^	29/81 (36%)	10/87 (12%)	< 0.001
Corticosteroids (high dose)*	1/81 (1%)	2/87 (2%)	0.60
**ARDS treatment during ICU stay**			
Corticosteroids (any dose)*	37/81 (46%)	35 /87 (40%)	0.48
Corticosteroids (low dose)*^#^	33/81 (41%)	25/87 (29%)	0.10
Corticosteroids (high dose)*	3/81 (4%)	10/87 (12%)	0.06
Prone position	34 (42%)	75 (83%)	< 0.001
Neuromuscular blockade	53 (65%)	83 (92%)	< 0.001
Inhaled nitric oxide	10 (12%)	31 (34%)	0.01
Extra-corporeal membrane oxygenation	9 (11%)	23 (26%)	0.014
**ICU-acquired infections**			
First VAP	36 (44%)	58 (64%)	0.007
Number of days of mechanical ventilation before first VAP	7 [5–9]	8 [5–12]	0.89
Number of VAP during ICU	0 [0–1]	1 [0–2]	< 0.001
Recurrent VAP	10 (12%)	22 (25%)	0.36
MDR VAP during ICU stay	9 (11%)	21 (23%)	0.03
ESBL PE VAP	9 (11%)	18 (20%)	0.10
MRSA VAP	0	1 (1%)	0.99
CRE VAP	0	3 (3%)	0.095
Sampling frequency (number/day of MV)	0.23 [0.14–0.37]	0.32 [0.20–0.38]	0.03
**Organ support and outcome during ICU stay**			
Subglottic secretion drainage	26 (32%)	42 (47%)	0.045
Renal replacement therapy during ICU stay	28 (34%)	30 (33%)	0.91
Norepinephrine, *n* (%)	66 (81%)	67 (74%)	0.34
ICU length of stay among survivors, days	15 [10–20]	30 [19–45]	< 0.001
Successful mechanical ventilation weaning	54 (66%)	46 (51%)	0.05
Death at day 28	25 (31%)	36 (40%)	0.19
Death in the ICU	27 (33%)	37 (41%)	0.27
Still in ICU or in weaning center (until May 28th, 2020)	0	8 (9%)	0.007

*VAP* ventilator-associated pneumonia, *COPD* chronic obstructive pulmonary disease, *HIV* human immunodeficiency virus, *SAPS II* Simplified Acute Physiology Score II, *SOFA* sequential organ failure assessment, *ICU* intensive care unit, *MDR* multi-drug resistant, *ESBL-PE* extended-spectrum β-lactamase-producing *Enterobacteriaceae*, *MRSA* methicillin-resistant *Staphylococcus aureus*, *CRE* carbapenem-resistant *Enterobacteriaceae*, *MV* mechanical ventilation

^*^Four missing values because two patients received dexamethasone or placebo in a randomized controlled trial

^#^Less than 1 mg/kg of prednisone or equivalent

### Coinfection at ICU admission

There were significantly fewer documented bacterial coinfections in C-ARDS than in NC-ARDS (Table 1). The microorganisms involved in bacterial coinfection at ICU admission are reported in Additional file 2: Table S1. The types of isolated microorganisms differed between the groups with fewer Gram-positive cocci in C-ARDS than in NC-ARDS, 4/14 (29%) vs. 23/39 (59%), *p* = 0.05, but similar Gram-negative bacilli [9/14 (64%) vs. 19/39 (49%), *p* = 0.32].

### VAP occurrence and risk factors

**Fig. 1:**
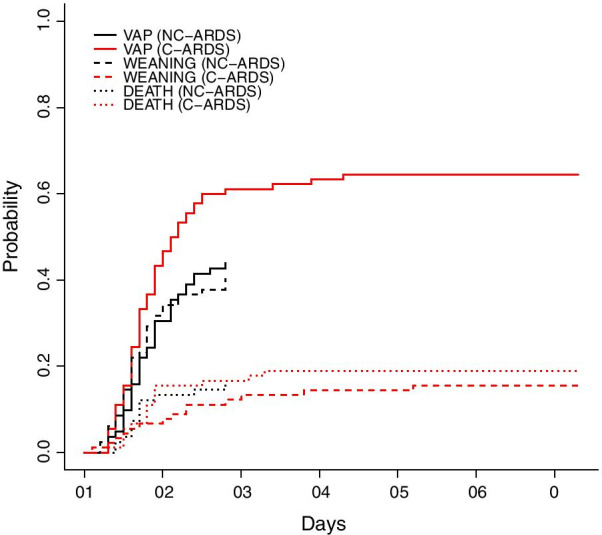
Cumulative probability of ventilator-associated pneumonia (VAP) in C-ARDS (red) and NC-ARDS (blue) patients. For analysis purpose, time from intubation to VAP (continuous line), to death (dotted line), and to weaning (dashed line) were handled as competing risks

At day 28 of ICU admission, significantly more patients developed at least one VAP episode in C-ARDS group than in NC-ARDS group: 56 (62%) vs. 35 (43%), *p* = 0.016. The mechanical ventilation lasted longer in C-ARDS than in NC-ARDS group: 16.5 [9.0–28.8] vs 9.0 [6.0–17.3] days, *p* < 0.0001. Fine and Gray model results showed that VAP probability was significantly higher in C-ARDS group after adjusting for death and ventilator weaning [sub-hazard ratio = 1.72 (1.14–2.57), *p* < 0.01, Fig. 1]. Conversely, the probability of successful ventilator weaning was significantly reduced in C-ARDS group after adjusting for VAP and death as competing events [sub-hazard ratio = 0.34 (0.19–0.63), *p* < 0.001]; the probability of death was similar in both groups [sub-hazard ratio = 1.18 (0.58–2.41), *p* = 0.64, Fig. 1]. These results persisted after matching for ARDS severity (mild, moderate or severe) and diabetes mellitus, with a higher VAP probability (after adjusting for death and ventilator weaning) and a lower weaning probability (after adjusting for VAP and death) in C-ARDS group [sub-hazard ratio of 1.74 (1.09–2.80), *p* = 0.02, and 0.37 (0.20–0.70), *p* < 0.01, respectively]. Risk factors for developing VAP were tested by univariate analysis in Additional file 3: Table S2. The factors associated with VAP occurrence, as shown by multivariable analysis (Additional file 4: Table S3), were C-ARDS [OR = 2.1 (1.1–4.0), *p* = 0.02] and male gender [OR = 2.2 (1.04–4.5), *p* = 0.04]. These results were similar by competing risk analysis: VAP probability was significantly higher in C-ARDS group as compared to NC-ARDS [sub-hazard ratio = 1.58 (1.05–2.39), *p* = 0.03] and in males as compared to females [sub-hazard ratio = 1.72 (1.03–2.88), *p* = 0.04], while adjusting for death and ventilator weaning.

### VAP documentation and management

The most commonly isolated microorganisms in both groups with VAP were *Enterobacteriaceae*, which were more common in C-ARDS than in NC-ARDS group: *n* = 42 (72%) vs. 17 (47%), *p* = 0.01 at the first VAP episode (Table 2). MDR bacteria (all were ESBL-PE except one CRE New Delhi metallo-β-lactamase (NDM)) were retrieved in eleven (20%) C-ARDS and seven (19%) NC-ARDS patients during the first VAP episode (*p* = 0.57). However, the incidence of MDR VAP was significantly higher in C-ARDS than NC-ARDS during the entire ICU stay: 21 (23%) vs. 9 (11%), *p* = 0.03. Three C-ARDS patients had MDR VAP caused by CRE [two NDM and one oxacillinase-48 (OXA-48) producing *Enterobacteriaceae*, all without ESBL coproduction], 18 had ESBL-PE, and one had polymicrobial VAP caused by methicillin-resistant *Staphylococcus aureus* and ESBL-PE. In NC-ARDS patients, nine had their VAP caused by ESBL-PE. All patients received antibiotics during their ICU stay. Rate of administering Carbapenem for the first VAP was 16 (18%) in C-ARDS vs. 9 (11%) in NC-ARDS, *p* = 0.21. The three mostly used antibiotics were amoxicillin/clavulanic acid, third-generation cephalosporin, and piperacillin/tazobactam in NC-ARDS group, versus third-generation cephalosporin, carbapenem, and aminoglycoside in C-ARDS group. Carbapenem was more used in C-ARDS than in NC-ARDS patients: 48 (53%), vs 21 (26%), *p* < 0.01 (Additional file 5: Table S4). Consuming alcohol-based handrub liquid in the ICU reached 135 mL per patient-day for the NC-ARDS study period versus 522 mL per patient-day for the C-ARDS study period.

**Table 2:** Microorganisms involved in first ventilator-associated pneumonia in patients with acute respiratory distress related to Coronavirus disease 19 (C-ARDS) or other viruses (NC-ARDS)

Microorganisms	NC-ARDS (*n* = 36)	C-ARDS (*n* = 58)
**Gram-negative bacilli**		
*Haemophilus sp*	4 (11%)	0
**Enterobacteriaceae**	17 (47%)	42 (72%)
* Enterobacter sp*	4 (11%)	23 (40%)
* Klebsiella pneumoniae*	6 (17%)	4 (7%)
* Citrobacter sp*	1 (3%)	2 (4%)
* Escherichia coli*	4 (11%)	10 (17%)
* Hafnia*	0	2 (4%)
* Morganella morganii*	1 (3%)	0
* Serratia*	2 (6%)	1 (2%)
* Proteus*	0	4 (7%)
Extended-spectrum beta-lactamase-producing enterobacteriaceae	7 (19%)	10 (18%)
Carbapenem-resistant enterobacteriaceae	0	1 (2%)
**Non-fermenting gram-negative bacilli**	20 (56%)	24 (41%)
* Acinetobacter sp*	1 (3%)	1 (2%)
* Pseudomonas sp*	17 (47%)	16 (28%)
* Burkholderia Cepacia*	0	1 (2%)
* Stenotrophomonas maltophilia*	2 (6%)	3 (5%)
**Gram-positive bacteria**	0	4 (3%)
* Streptococcus pneumoniae*	0	0
* Others Streptococcus* sp	0	2 (4%)
Methicillin-sensitive *Staphylococcus aureus*	0	2 (4%)
Methicillin-resistant *Staphylococcus aureus*	0	0
* Enterococcus faecalis*	0	1 (2%)
**Polymicrobial**	4 (11%)	13 (22%)
**Diagnostic sampling techniques**		
Bronchoalveolar lavage	4 (11%)	3 (5%)
Blind protected telescope catheter	32 (89%)	55 (95%)

The total number of microorganisms is greater than 100% because more than one microorganism may be retrieved from a given respiratory sample

### Invasive aspergillosis

Diagnostic criteria for invasive pulmonary aspergillosis according to IAPA case definition, crude AspICU definition, and modified AspICU definition are shown in Table 3. There was no proven aspergillosis case in the entire study. Probable aspergillosis (as per IAPA case definition) and putative aspergillosis (as per crude and modified AspICU criteria) were less common in C-ARDS than in NC-ARDS patients (Table 3). According to AspICU algorithm, there was no difference in *Aspergillus* colonization between C-ARDS and NC-ARDS patients. Univariate analysis of risk factors for developing invasive pulmonary aspergillosis is reported in Additional file 6: Table S5. The factors associated with invasive pulmonary aspergillosis as tested by multivariable analysis (Additional file 7: Table S6) were immunodepression [OR = 3.6 (1.4–9.1), *p* = 0.01] and having influenza, which fell short of statistical significance [OR = 2.5 (0.88–6.4), *p* = 0.052). Invasive pulmonary aspergillosis was associated with ICU mortality (Additional file 6: Table S5).

**Table 3:** Diagnostic criteria of invasive aspergillosis in patients with acute respiratory distress syndrome related to Coronavirus disease 19 (C-ARDS) or other viruses (NC-ARDS)

	NC-ARDS (*n* = 82)	C-ARDS (*n* = 90)	*p* value
**IAPA case definition**			
***Proven invasive pulmonary aspergillosis:*** Biopsy or brush specimen of airway plaque, pseudomembrane, or ulcer showing hyphal elements and *Aspergillus* growth on culture or positive *Aspergillus* PCR on tissue. Lung biopsy showing invasive fungal elements and Aspergillus growth on culture or positive Aspergillus PCR on tissue	0	0	
***Probable invasive pulmonary aspergillosis***	17 (21%)	7 (8%)	0.01
Probable invasive pulmonary aspergillosis diagnosed at ICU admission	9 (53%)	3 (43%)	
Time from admission to diagnosis of probable pulmonary aspergillosis during ICU stay, days	10 [7–14]	6 [5–11]	
***Aspergillus tracheobronchitis***	1 (1%)	1 (1%)	0.95
Airway plaque, pseudomembrane, or ulcer	3/49	1/24	
**IAPA in patients without documented Aspergillus tracheobronchitis**	16 (20%)	6 (7%)	0.01
Pulmonary infiltrate	82	90	
Cavitating infiltrate (not attributed to another cause)	1	1	
Serum GM index > 0.5	6/40	5/88	
BAL GM index ≥ 1.0	5/31	0/0	
Positive BAL culture	10/50	4/24	
**Crude AspICU criteria**			
***Proven invasive pulmonary aspergillosis:***	0	0	
***Putative invasive pulmonary aspergillosis***	12 (15%)	2 (2%)	0.003
1. Aspergillus-positive lower respiratory tract specimen culture*	17	6	
2. Compatible signs and symptoms	16	6	
3. Abnormal medical imaging by portable chest X-ray or CT-scan	14	5	
4a. Host risk factors	9	0	
4b. Semiquantitative Aspergillus-positive culture of BAL fluid (+ or ++), without bacterial growth together with a positive cytological smear showing branching hyphae	8	2	
***Colonization***	5 (6%)	4 (5%)	0.63
**Modified AspICU criteria**			
***Proven invasive pulmonary aspergillosis:***	0	0	
***Putative invasive pulmonary aspergillosis***	15 (18%)	6 (7%)	0.02
***Mycological criteria***	18	7	
Histopathology or direct microscopic evidence of dichotomous septate hyphae with positive culture for Aspergillus on tissue	0	0	
Serum GM index > 0.5	6/40	5/88	
BAL GM index ≥ 1.0	5/31	0/0	
Positive BAL culture	10/50	4/24	
Compatible signs and symptoms^§^	17/18	6/7	
Abnormal medical imaging by portable chest X-ray or CT-scan^§^	16/18	7/7	
**Other diagnostic criteria**			
Aspergillus PCR on lower respiratory tract: positive cases / performed cases	0 /7 (0%)	16 /81(20%)	
1,3-β-D-glucan: positive cases /performed cases	8/23(35%)	9 /88(10%)	

^*^Entry criterion for Crude AspICU criteria. Data are *n* (%) or *n*/*N* (%).* BAL* bronchoalveolar lavage

^§^Among patients with positive mycological criteria

## Discussion

In this study of patients having viral ARDS, we have evidenced the following findings: i) Fewer documented bacterial coinfections in C-ARDS than in NC-ARDS at ICU admission; ii) a higher incidence of VAP and MDR VAP in C-ARDS than in NC-ARDS; and iii) a lower risk of putative invasive pulmonary aspergillosis in the C-ARDS group.

### Coinfection at ICU admission

There were significantly fewer documented bacterial coinfections in C-ARDS than in NC-ARDS. Bacterial coinfection rate in NC-CARDS group is in accordance with previous studies on ARDS secondary to influenza. Bacterial coinfection rate at ICU admission is reported in less than 10% in C-ARDS cases [22], except in small series using multiplex PCR assay [23, 24].

### Mechanical ventilation

The high incidence of VAP found in our study is in accordance with the selected population of ARDS (see flowchart), as reported in previous studies [11, 21, 25, 26]. Using ARDS as a selection criteria allowed inclusion of a relatively homogenous group of patients with reduction of potential bias, given that ARDS is a known major risk factor for VAP. In our cohort, all patients with SARS-CoV-2 pneumonia requiring mechanical ventilation fulfilled ARDS criteria. This finding may be ascribable to the virulence of SARS-CoV-2 and/or to the fact that some intermediate care units have been deployed upstream the ICU for the care of patients not requiring immediate intubation during the pandemic. Invasive mechanical ventilation is a cornerstone in the development of VAP. The duration of mechanical ventilation was twice longer in C-ARDS than in NC-ARDS patients, with more recurrent VAP episodes in the former group. Strategies aimed at avoiding intubation, such as continuous positive airway pressure [27], high-flow nasal oxygen [28], or awake prone position in spontaneously breathing patients [29] should be further explored. Sedation protocols should also be optimized to reduce the duration of mechanical ventilation. SARS-CoV-2 infection was associated with encephalopathy, agitation, and confusion [30]. Our competing risk model yielded a reduced probability of ventilator weaning and a higher probability of VAP in C-ARDS patients whenever adjusted for ventilator weaning. Other factors may influence the risk for VAP in C-ARDS, like infectious process or infection control.

Low dose dexamethasone is reported as the first drug to improve survival in COVID-19 pneumonia [31]. In our work, 36% of NC-ARDS and 12% of C-ARDS patients were on low-dose corticosteroid at ICU admission, yet did not increase their risk for VAP. A recent randomized controlled study on dexamethasone treatment in ARDS did not show more ICU acquired infections [32]. Seven patients in the C-ARDS group received tocilizumab. Anti-inflammatory treatment may be associated with the development of bloodstream infection or late onset infections in recent studies [33, 34].

### Infection control

Hand hygiene compliance rate was not evaluated by internal audits in our ICU during COVID-19 outbreak. Consumption of alcohol-based handrub liquid was 135 mL per patient-day before the outbreak versus 522 mL per patient-day during COVID 19 period. This increase is probably due to the increased number of health care workers coming to reinforce our team. We do not know if the overall increase in alcohol-based handrub solution consumption was associated with a better hand hygiene compliance. Previous studies showed that during routine clinical care of patients with MDR bacteria, health care workers often contaminate protective gowns and gloves [35]. Moreover, teams dedicated to some procedures like prone positioning were created to decrease nurse work strain, but their transversal nature may have increased the risk of cross contamination. Now more than ever, health systems should continue investing in their infection prevention programs, beyond the current pandemic.

### Invasive aspergillosis

Severe influenza infection has been associated with invasive pulmonary aspergillosis. IAPA case definition and modified *AspICU* algorithm, specifically designed for severe influenza, were used in this study to assess the role of invasive aspergillosis in C-ARDS. A comprehensive diagnostic approach for invasive aspergillosis was implemented in C-ARDS using a systematic serum galactomannan test, PCR and culture of lower respiratory tract secretions for *Aspergillus* species, and 1,3-β-D-glucan. Four patients with putative aspergillosis had no previous risk factors, suggesting that C-ARDS is a host factor for invasive aspergillosis. However, the incidence of invasive aspergillosis was significantly lower in C-ARDS than in NC-ARDS and was consistent with previously reported patients with bacterial ARDS [36]. Pre-pandemic environmental air sampling found *Aspergillus* conidia in our ICU rooms. Laminar air flow unit and high-efficiency particulate air filters were installed during the outbreak which may have decrease invasive *Aspergillus* nosocomial infection [37] and explain the lower rate of invasive aspergillosis in C-ARDS in our work, but half of invasive aspergillosis cases were diagnosed at ICU admission. The lack of galactomannan in BAL performed in C-ARDS patients may underestimate invasive aspergillosis using IAPA case definition and modified *AspICU* algorithm, but putative aspergillosis was less common in C-ARDS using crude AspICU criteria. Bartoletti et al. [38] found a higher incidence of invasive pulmonary aspergillosis in COVID-19 patients mostly treated with high doses corticosteroids and tocilizumab. In our cohort, NC-ARDS patients were often immunosuppressed with more blood malignancies and corticosteroid, which are known risk factors for invasive aspergillosis in patients with ARDS or severe influenza [16, 36]. These underlying diseases may at least in part explain the higher risk of invasive aspergillosis in this group.

### Strengths and limitations

The strengths of our study come from the detailed description of VAP and the use of competing risk models (cumulative incidence function of Gray model) to properly estimate the effect of COVID-19 on VAP risk, after adjustment on death and ventilator weaning as competing events.

Our study has some limitations. First, due to its monocentric design, our results may not be applicable on other centers. The risks of VAP may vary between centers in parallel with the variation in infection prevention measures and health crisis preparedness strategies. Clinical wards air and contact surfaces, sources of pathogenic fungi, may highly vary between ICUs, especially during a crisis. Second, the study period used to recruit the cohort was long (11 years), which is a major limitation. The lower incidence of ARDS in patients with non-COVID viral pneumonia may be ascribable to a lower virulence and transmissibility of non-SARS-CoV-2 respiratory viruses as compared to SARS-CoV-2 [39]. However, our management protocol for ARDS did not significantly change during the study period. Third, we found different baseline characteristics between groups. C-ARDS patients were mostly males [40, 41], a known risk factor of VAP; however, the latter association with C-ARDS persisted in the multivariable analysis and in matched analysis. Fourth, it might be difficult to interpret chest X-ray because of preexisting parenchymal injury in ARDS patients [42]. The microbiological investigation on lower respiratory tract samples is currently the main diagnostic tool of VAP in C-ARDS and NC-ARDS [43]. The higher respiratory sampling in C-ARDS patients may have theoretically contributed to an overestimation of the VAP frequency in this group, but VAP was first clinically suspected if any of its classical criteria happened and sampling was then performed to confirm VAP. We cannot exclude that respiratory deterioration-labeled VAP was to some extent relative to progression of COVID-19 with a bystander positive bacterial culture.

## Conclusions

In this retrospective study, we have observed a higher incidence of VAP and MDR VAP in C-ARDS as compared with NC-ARDS patients. Further, probably multicenter, research work are needed to confirm this association.

## Supplementary Information

**Additional file 1**. Figure S1 (online supplement): Flowchart of the study. ARDS denotes Acute Respiratory Distress Syndrome; C-ARDS denotes COVID-19-related ARDS; NC-ARDS denotes non-COVID-19 related ARDS.

**Additional file 2**. Table S1. Microorganisms involved in bacterial coinfection documented at intensive care unit admission in patients with acute respiratory disease related to Coronavirus disease 19 (C-ARDS) or other viruses (NC-ARDS).

**Additional file 3**. Table S2. Univariate analysis of variables associated with ventilator-associated pneumonia (VAP) in patients with acute respiratory distress syndrome related to Coronavirus disease 19 (C-ARDS) or other viruses (NC-ARDS).

**Additional file 4**. Table S3. Multivariable logistic regression testing factors associated with ventilator-associated pneumonia in patients with acute respiratory distress syndrome related to Coronavirus disease 19 (C-ARDS) or other viruses (NC-ARDS).

**Additional file 5**. Table S4. Antibiotics use during intensive care unit stay in patients with acute respiratory disease syndrome related to Coronavirus disease 19 (C-ARDS) or other viruses (NC-ARDS).

**Additional file 6**. Table S5. Univariate analysis of factors associated with invasive pulmonary aspergillosis (Influenza-Associated Pulmonary Aspergillosis case definition) in patients with acute respiratory distress syndrome related to Coronavirus disease 19 (C-ARDS) or other viruses (NC-ARDS).

**Additional file 7**. Table S6. Multivariable logistic regression of factors associated with invasive pulmonary aspergillosis (Influenza-Associated Pulmonary Aspergillosis case definition) in patients with acute respiratory distress syndrome related to Coronavirus disease 19 (C-ARDS) or other viruses (NC-ARDS).

## Data Availability

The datasets supporting the conclusions are included within the article and supplementary data.

## Abbreviations

VAP: Ventilator-associated pneumonia
C-ARDS: Coronavirus disease 19-related acute respiratory distress syndrome
NC-ARDS: Non-Coronavirus disease 19 viral ARDS
MDR: Multi-drug-resistant bacteria
ESBL-PE: Extended-spectrum β-lactamase-producing *Enterobacteriaceae*
