# Correction to: Risks of ventilator-associated pneumonia and invasive pulmonary aspergillosis in patients with viral acute respiratory distress syndrome related or not to Coronavirus 19 disease

**DOI:** 10.1186/s13054-021-03517-5

**Published:** 2021-03-22

**Authors:** Keyvan Razazi, Romain Arrestier, Anne Fleur Haudebourg, Brice Benelli, Guillaume Carteaux, Jean‑Winoc Decousser, Slim Fourati, Paul Louis Woerther, Frederic Schlemmer, Anais Charles-Nelson, Francoise Botterel, Nicolas de Prost, Armand Mekontso Dessap

**Affiliations:** 1 AP-HP (Assistance Publique-Hôpitaux de Paris), Hôpitaux universitaires Henri Mondor, DMU Médecine, Service de Médecine Intensive Réanimation, 94010 Créteil, France; 2 UPEC (Université Paris Est Créteil), Faculté de Santé de Créteil, IMRB, GRC CARMAS, 94010 Créteil, France; 3 UPEC (Université Paris Est), INSERM, Unité U955, 94010 Créteil, France; 4 AP-HP (Assistance Publique-Hôpitaux de Paris), Hôpitaux universitaires Henri Mondor, Contrôle, Epidémiologie et Prévention de l’Infection, CEPI, 94010 Créteil, France; 5 AP-HP (Assistance Publique-Hôpitaux de Paris), Hôpitaux universitaires Henri Mondor, Département de Virologie, Bactériologie, Parasitologie-Mycologie, 94010 Créteil, France; 6 UPEC (Université Paris Est), EA 7380 Dynamic, Ecole nationale vétérinaire d’Alfort, USC Anses, Créteil, France; 7 AP-HP (Assistance Publique-Hôpitaux de Paris), Hôpitaux universitaires Henri Mondor, DHU A-TVB, Unité de Pneumologie, 94010 Créteil, France; 8 AP-HP (Assistance Publique Hôpitaux de Paris), Hôpital européen Georges Pompidou, Unité d’Épidémiologie et de Recherche Clinique, INSERM, Centre d’Investigation Clinique1418, module Épidémiologie Clinique, Paris, France

## Correction to: Crit Care (2020) 24:699 https://doi.org/10.1186/s13054-020-03417-0

Following publication of the original article [1], the authors identified an error in Fig. 1. The correct figure is given hereafter.

The correct Fig. 1 is:

**Fig. 1:**
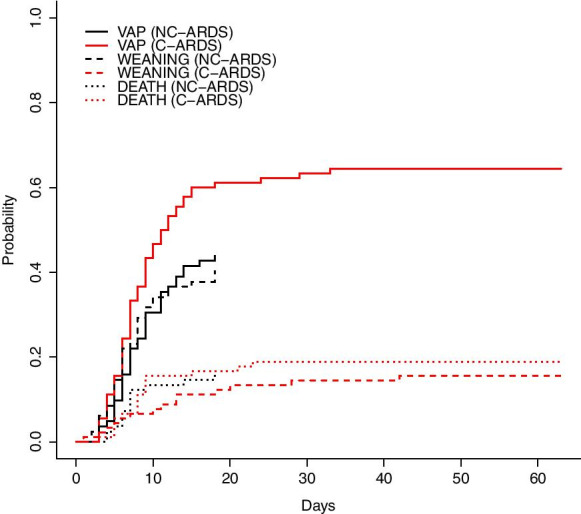
Cumulative probability of ventilator-associated pneumonia (VAP) in C-ARDS (red) and NC-ARDS (black) patients. For analysis purpose, time from intubation to VAP (continuous line), to death (dotted line), and to weaning (dashed line) were handled as competing risks

All the changes requested are implemented in this correction and the original article [1] has been corrected.
