# Supplementary material for: Risks of ventilator-associated pneumonia and invasive pulmonary aspergillosis in patients with viral acute respiratory distress syndrome related or not to Coronavirus 19 disease
Source: Crit Care. 2020 Dec 18;24:699. doi: 10.1186/s13054-020-03417-0 (PMC7747772; doi:10.1186/s13054-020-03417-0)
Supplement: Supplementary file 1 — Additional file 1. Figure S1 (online supplement): Flowchart of the study. ARDS denotes Acute Respiratory Distress Syndrome; C-ARDS denotes COVID-19-related ARDS; NC-ARDS denotes non-COVID-19 related ARDS. [file 13054_2020_3417_MOESM1_ESM.pptx]

## Slide 1
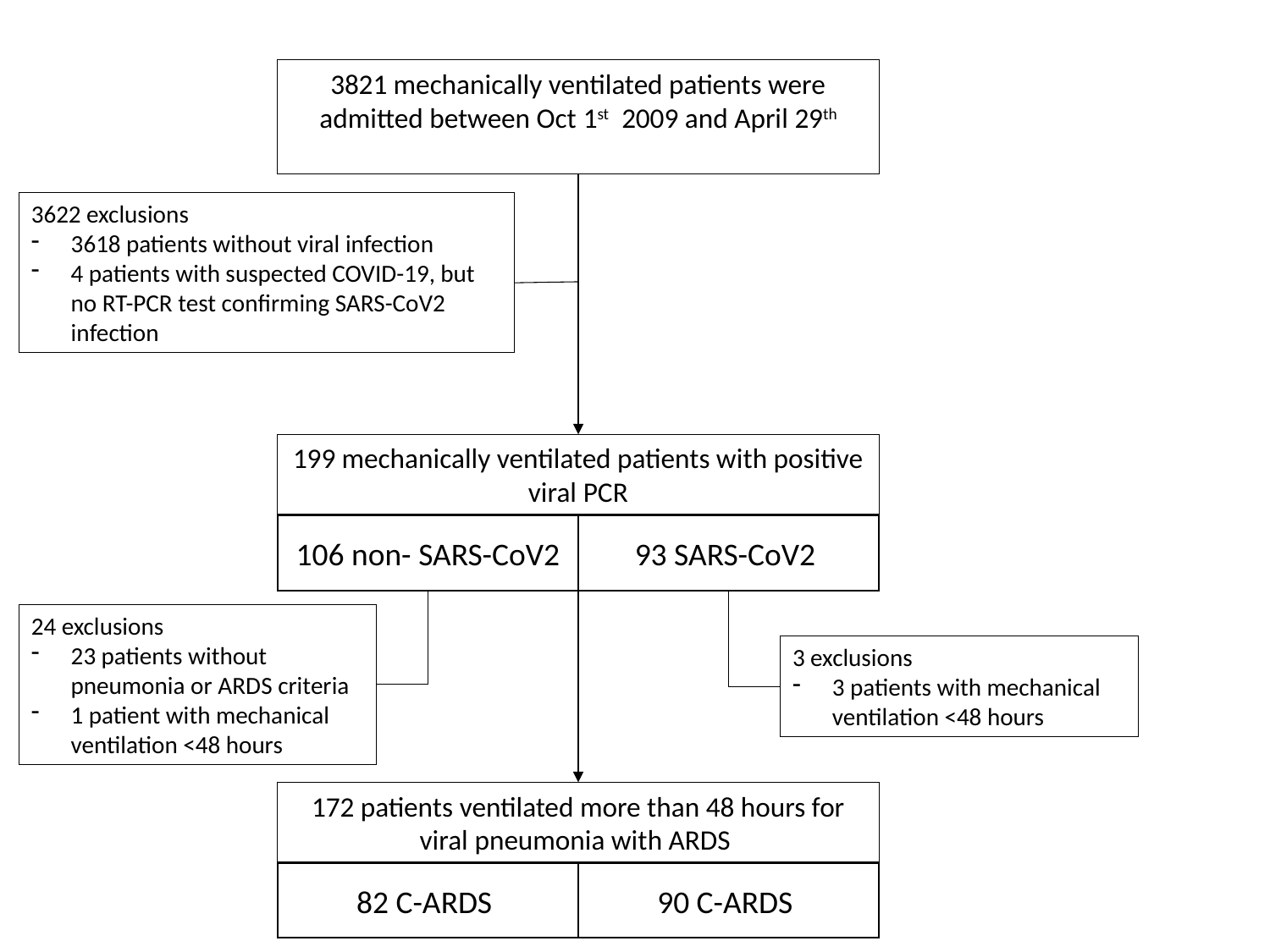

3821 mechanically ventilated patients were admitted between Oct 1st 2009 and April 29th
3622 exclusions
3618 patients without viral infection
4 patients with suspected COVID-19, but no RT-PCR test confirming SARS-CoV2 infection
199 mechanically ventilated patients with positive viral PCR
106 non- SARS-CoV2
93 SARS-CoV2
24 exclusions
23 patients without pneumonia or ARDS criteria
1 patient with mechanical ventilation <48 hours
3 exclusions
3 patients with mechanical ventilation <48 hours
172 patients ventilated more than 48 hours for viral pneumonia with ARDS
82 C-ARDS
90 C-ARDS
