# Supplementary material for: Risks of ventilator-associated pneumonia and invasive pulmonary aspergillosis in patients with viral acute respiratory distress syndrome related or not to Coronavirus 19 disease
Source: Crit Care. 2020 Dec 18;24:699. doi: 10.1186/s13054-020-03417-0 (PMC7747772; doi:10.1186/s13054-020-03417-0)
Supplement: Supplementary file 2 — Additional file 2. Table S1. Microorganisms involved in bacterial coinfection documented at intensive care unit admission in patients with acute respiratory disease related to Coronavirus disease 19 (C-ARDS) or other viruses (NC-ARDS). [file 13054_2020_3417_MOESM2_ESM.docx]

**Table S1. Micro-organisms involved in bacterial co-infection documented at intensive care unit admission in patients with acute respiratory disease related to Coronavirus disease 19 (C-ARDS) or other viruses (NC-ARDS).**

|  | **NC-ARDS** | **C-ARDS** |
| --- | --- | --- |
| **Micro-organisms** | **(n=39)** | **(n=14)** |
| **GRAM NEGATIVE BACILLI** | 19 (49%) | 9 (64%) |
| *Haemophilus sp* | 8 (21%) | 0 |
| **Enterobacteriaceae** |  |  |
| *Enterobacter sp* | 2 (5%) | 1 (7%) |
| *Klebsiella pneumoniae* | 4 (11%) | 1 (7%) |
| *Citrobacter sp* | 0 | 1 (7%) |
| *Escherichia coli* | 1 (3%) | 0 |
| *Hafnia* | 0 | 2 (14%) |
| *Morganella morganii* | 0 | 0 |
| *Serratia* | 0 | 0 |
| *Proteus* | 0 | 0 |
| Extended-spectrum beta-lactamase-producing Enterobacteriaceae | 1 (3%) | 0 |
| Carbapenem-Resistant Enterobacteriaceae | 0 | 0 |
| **Non-fermenting gram-negative bacilli** |  |  |
| *Acinetobacter sp* | 1 (3%) | 0 |
| *Pseudomonas sp* | 3 (8%) | 3 (21%) |
| *Burkholderia Cepacia* | 0 | 1 (7%) |
| *Stenotrophomonas maltophilia* | 0 | 0 |
| **GRAM POSITIVE BACTERIA** | 23 (59%) | 4 (29%) |
| *Streptococcus pneumoniae* | 10 (%) | 1 (7%) |
| *Others Streptococcus* sp | 3 (8%) | 1 (7%) |
| Methicillin-sensitive *Staphylococcus aureus* | 9 (23%) | 3 (21%) |
| Methicillin-resistant *Staphylococcus aureus* | 1 (3%) | 0 |
| *Enterococcus faecalis* | 0 | 0 |
| **OTHER** |  |  |
| *Branhamella catarrhalis* | 1 (3%) | 2 (14%) |
| **POLYMICROBIAL** | 4 (10%) | 2 (14%) |
| **Specimen collection and laboratory testing** |  |  |
| Pneumococcal urinary antigen test | 7/73 | 0/80 |
| Legionella urinary antigen test | 0/73 | 0/87 |
| **First sample positive/ performed** |  |  |
| Sputum | 2/4 | 5/13 |
| Blind protected telescope catheter | 30/60 | 6/72 |
| Endotracheal Aspiration | 2/3 | 2/2 |
| Bronchoalveolar lavage | 5/15 | 1/1 |

The total number of micro-organisms is greater than 100% because more than one micro-organism may be retrieved from a given respiratory sample.
