# Supplementary material for: Risks of ventilator-associated pneumonia and invasive pulmonary aspergillosis in patients with viral acute respiratory distress syndrome related or not to Coronavirus 19 disease
Source: Crit Care. 2020 Dec 18;24:699. doi: 10.1186/s13054-020-03417-0 (PMC7747772; doi:10.1186/s13054-020-03417-0)
Supplement: Supplementary file 3 — Additional file 3. Table S2. Univariate analysis of variables associated with ventilator-associated pneumonia (VAP) in patients with acute respiratory distress syndrome related to Coronavirus disease 19 (C-ARDS) or other viruses (NC-ARDS). [file 13054_2020_3417_MOESM3_ESM.docx]

**Table S2. Univariate analysis of variables associated with ventilator associated pneumonia (VAP) in patients with acute respiratory distress syndrome related to Coronavirus disease 19 (C-ARDS) or other viruses (NC-ARDS).**

| **Variables** | **No VAP**  **(n=78)** | **VAP**  **(n=94)** | **P value** |
| --- | --- | --- | --- |
| Age median [IQR] | 62 [56-71] | 61 [52-69] | 0.38 |
| Male gender | 51(65%) | 77 (82%) | 0.01 |
| **Medical History** |  |  |  |
| Mc Cabe’s classification  No underlying disease  Ultimately fatal  Rapidly fatal disease | 53 (68%)  20 (26%)  5 (6%) | 70 (75%)  16 (17%)  8 (9%) | 0.36 |
| Charlson Comorbidity Index | 2 [0-3] | 1 [0-2] | 0.22 |
| Diabetes Mellitus | 28 (36%) | 34 (36%) | 0.97 |
| Congestive heart failure (NYHA 3-4) | 9 (12%) | 4 (4%) | 0.07 |
| Supraventricular arrhythmia | 9 (12%) | 11 (12%) | 0.97 |
| Hypertension | 45 (58%) | 50 (53%) | 0.55 |
| COPD | 11 (14%) | 8 (9%) | 0.24 |
| Chronic renal failure | 11 (14%) | 14 (15%) | 0.88 |
| Dialysis | 2 (3%) | 3 (3%) | 0.80 |
| Stroke | 5 (6%) | 4 (4%) | 0.53 |
| Liver cirrhosis (Child C) | 0 (0%) | 1(1%) | 0.36 |
| Smoking | 23(30%) | 24 (26%) | 0.56 |
| Immunodepression | 24 (31%) | 32 (34%) | 0.65 |
| ***Clinical characteristics upon ICU admission*** |  |  |  |
| SAPS II | 44 [35-57] | 38 [30-49] | 0.04 |
| Baseline SOFA— median [IQR] | 8 [5-11] | 7 [4-8] | 0.01 |
| PaO2/FiO2 (mmHg) median [IQR] | 143 [103-183] | 123 [93-188] | 0.28 |
| Norepinephrine, n (%) | 48 (62%) | 37 (39%) | 0.004 |
| Lymphocyte count (× 10^9^/L) | 0.8 [0.5-1.3] | 0.7 [0.4-1.0] | 0.26 |
| Bacterial coinfection | 30 (39%) | 23 (25%) | 0.048 |
| Influenza | 25 (32%) | 25 (27%) | 0.43 |
| C-ARDS | 32 (41%) | 58 (62%) | 0.007 |
| **Treatment at ICU admission** |  |  |  |
| Antibiotic treatment for 48h or more following intubation | 76 (97%) | 85 (90%) | 0.11 |
| Subglottic secretion drainage | 27 (35%) | 41 (44%) | 0.23 |
| Neuromuscular blockade | 41 (53%) | 48 (51%) | 0.85 |
| Prone position | 20 (26%) | 33 (35%) | 0.18 |
| Extra corporeal membrane oxygenation | 3 (4%) | 9 (10%) | 0.14 |
| Corticosteroids (any dose) * | 19/77 (25%) | 23/91 (25%) | 0.93 |
| Corticosteroids (low dose) *# | 18/77 (23%) | 21/91 (23%) | 0.96 |
| Corticosteroids (high dose) * | 1/77 (1%) | 2/91 (2%) | 0.99 |
| **Outcome** |  |  |  |
| ICU length of stay | 10 [7-16] | 23 [17-38] | <0.001 |
| Among survivors | 13 [8-18] | 30 [20-46] | <0.001 |
| Death at day 28 | 31 (40%) | 30 (32%) | 0.29 |
| Death in ICU | 31(40%) | 33 (35%) | 0.53 |

Abbreviations: VAP ventilator-associated pneumonia, COPD=chronic obstructive pulmonary disease, SAPS II =Simplified Acute Physiology Score II, SOFA = sequential organ failure assessment, ICU= intensive care unit. * Four missing values because two patients received dexamethasone or placebo in a randomized controlled trial; #denotes less than 1 mg/kg of prednisone or equivalent
