# Supplementary material for: Risks of ventilator-associated pneumonia and invasive pulmonary aspergillosis in patients with viral acute respiratory distress syndrome related or not to Coronavirus 19 disease
Source: Crit Care. 2020 Dec 18;24:699. doi: 10.1186/s13054-020-03417-0 (PMC7747772; doi:10.1186/s13054-020-03417-0)
Supplement: Supplementary file 4 — Additional file 4. Table S3. Multivariable logistic regression testing factors associated with ventilator-associated pneumonia in patients with acute respiratory distress syndrome related to Coronavirus disease 19 (C-ARDS) or other viruses (NC-ARDS). [file 13054_2020_3417_MOESM4_ESM.docx]

**Table S3. Multivariable logistic regression testing factors associated with ventilator associated pneumonia in patients with acute respiratory distress syndrome related to Coronavirus disease 19 (C-ARDS) or other viruses (NC-ARDS).**

|  | **Missing**  **values, n (%)** | **Odd ratio (95% confidence interval), p value**  **by logistic regression** | |
| --- | --- | --- | --- |
|  |  | **Univariate** | **Multivariable** |
| Male gender | 0 | 2.4 (1.2-4.8), p=0.02 | 2.2 (1.04-4.5), p=0.04 |
| Congestive heart failure (NYHA 3-4) | 0 | 0.34 (0.10-1.15), p=0.08 | I/NR |
| SAPS II at ICU admission | 0 | 0.98 (0.97-0.99), p=0.04 | I/NR |
| Bacterial coinfection at ICU admission | 0 | 0.52 (0.27-1.00), p=0.048 | I/NR |
| C-ARDS | 0 | 2.3 (1.3-4.3), p=0.01 | 2.1 (1.1-4.0), p=0.02 |

I/NR=included, but not retained by the final model. Abbreviations: SAPS II= simplified acute physiology score. Among related univariate factors, only the most clinically relevant were entered into the regression model in order to minimize the effect of collinearity, as follows: SAPS II was selected among SAPS II, SOFA (Sequential Organ Failure Assessment), and norepinephrine. However, when SOFA was used instead of SAPS II, it yielded similar results. The multivariable model showed a good calibration as assessed by the Hosmer and Lemeshow goodness of fit test [χ^2^ (3 df) = 0.33, p=0.95].
