# Supplementary material for: Risks of ventilator-associated pneumonia and invasive pulmonary aspergillosis in patients with viral acute respiratory distress syndrome related or not to Coronavirus 19 disease
Source: Crit Care. 2020 Dec 18;24:699. doi: 10.1186/s13054-020-03417-0 (PMC7747772; doi:10.1186/s13054-020-03417-0)
Supplement: Supplementary file 5 — Additional file 5. Table S4. Antibiotics use during intensive care unit stay in patients with acute respiratory disease syndrome related to Coronavirus disease 19 (C-ARDS) or other viruses (NC-ARDS). [file 13054_2020_3417_MOESM5_ESM.docx]

**Table S4. Antibiotics use during intensive care unit stay in patients with acute respiratory disease syndrome related to Coronavirus disease 19 (C-ARDS) or other viruses (NC-ARDS).**

| **Antibiotics** | **NC-ARDS**  **(n=82)** | **C-ARDS**  **(n=90)** | **P value** |
| --- | --- | --- | --- |
| Aminopenicillins | 22 (27%) | 12 (13%) | 0.026 |
| amoxicillin/clavulanic acid | 28 (34%) | 26 (29%) | 0.46 |
| Third-generation cephalosporin | 48 (59%) | 77 (86%) | <0.001 |
| Piperacillin/Tazobactam | 52 (63%) | 44 (49%) | 0.055 |
| Cefepime/ Ceftazidime | 14 (17%) | 45 (50%) | <0.001 |
| Carbapenem | 21 (26%) | 48 (53%) | <0.001 |
| Aminoglycoside | 31 (38%) | 51 (57%) | 0.013 |
| Vancomycin | 5 (6%) | 18 (20%) | 0.007 |
| Fluoroquinolones | 16 (20%) | 22 (24%) | 0.44 |
