# Supplementary material for: Risks of ventilator-associated pneumonia and invasive pulmonary aspergillosis in patients with viral acute respiratory distress syndrome related or not to Coronavirus 19 disease
Source: Crit Care. 2020 Dec 18;24:699. doi: 10.1186/s13054-020-03417-0 (PMC7747772; doi:10.1186/s13054-020-03417-0)
Supplement: Supplementary file 6 — Additional file 6. Table S5. Univariate analysis of factors associated with invasive pulmonary aspergillosis (Influenza-Associated Pulmonary Aspergillosis case definition) in patients with acute respiratory distress syndrome related to Coronavirus disease 19 (C-ARDS) or other viruses (NC-ARDS). [file 13054_2020_3417_MOESM6_ESM.docx]

**Table S5. Univariate analysis of factors associated with invasive pulmonary aspergillosis (Influenza Associated Pulmonary Aspergillosis case definition) in patients with acute respiratory distress syndrome related to Coronavirus disease 19 (C-ARDS) or other viruses (NC-ARDS).**

|  | **Invasive pulmonary aspergillosis** | |  |
| --- | --- | --- | --- |
| **Variables** | **No**  **(n=148)** | **Yes**  **(n=24)** | **P value** |
| Age median [IQR] | 60 [52-69] | 68 [59-74] | 0.03 |
| Male gender | 108 (73%) | 20 (83%) | 0.28 |
| **Medical History** |  |  |  |
| Mc Cabe’s classification  No underlying disease  Ultimately fatal  Rapidly fatal disease | 113 (76%)  27 (18%)  8 (5%) | 10 (41%)  9 (38%)  5 (21%) | 0.001 |
| Charlson Comorbidity Index | 1 [0-2] | 3 [2-4] | 0.01 |
| Diabetes Mellitus | 54 (37%) | 8 (33%) | 0.77 |
| Congestive heart failure (NYHA 3-4) | 11 (7%) | 2 (8%) | 0.99 |
| Supraventricular arrhythmia | 17 (12%) | 3 (13%) | 0.99 |
| Hypertension | 82 (55%) | 13 (54%) | 0.91 |
| COPD | 17 (12%) | 2 (8%) | 0.99 |
| Chronic renal failure | 19 (13%) | 6 (25%) | 0.13 |
| Dialysis | 3 (2%) | 2 (8%) | 0.15 |
| Stroke | 8 (5%) | 1 (4%) | 0.99 |
| Liver cirrhosis (Child C) | 1 (1%) | 0 (0%) | 0.99 |
| Current smoking | 38 (26%) | 9 (38%) | 0.23 |
| Immunodepression | 41 (28%) | 15 (63%) | 0.001 |
| ***Clinical characteristics upon ICU admission*** |  |  |  |
| SAPS II | 39 [31-51] | 51 [35-67] | 0.12 |
| Baseline SOFA— median [IQR] | 7 [4-9] | 7 [5-11] | 0.34 |
| PaO2/FiO2 (mmHg) median [IQR] | 133 [96-185] | 147 [110-200] | 0.49 |
| Norepinephrine, n (%) | 46 (51%) | 9 (38%) | 0.21 |
| Lymphocyte count (× 10^9^/L) | 0.8 [0.4-1.2] | 0.4 [0.1-1.0] | 0.006 |
| Bacterial coinfection | 46 (31%) | 7 (29%) | 0.85 |
| Influenza | 38 (26%) | 12 (50%) | 0.015 |
| COVID-19 | 83 (56%) | 7 (29%) | 0.01 |
| Other viruses | 28 (19%) | 6 (25%) | 0.58 |
| **Treatment at admission** |  |  |  |
| Neuromuscular blockade | 80 (54%) | 9 (37%) | 0.13 |
| Prone position | 46 (31%) | 7 (29%) | 0.85 |
| Extra corporeal membrane oxygenation | 11 (7%) | 1 (4%) | 0.99 |
| Corticosteroids (any dose) * | 33/144 (23%) | 9 /24 (38%) | 0.13 |
| Corticosteroids (low dose) *# | 30/ 144 (21%) | 9/24 (38%) | 0.07 |
| Corticosteroids (high dose) * | 3/ 144 (2%) | 0/24 (0%) | 0.99 |
| **Outcome** |  |  |  |
| VAP | 79 (53%) | 15 (63%) | 0.41 |
| Death at day 28 | 49 (33%) | 12 (50%) | 0.11 |
| Death in ICU | 50 (34%) | 14 (58%) | 0.02 |

Abbreviations: VAP= ventilator-associated pneumonia, COPD=chronic obstructive pulmonary disease, SAPS II =Simplified Acute Physiology Score II, SOFA = sequential organ failure assessment, ICU= intensive care unit. * Four missing values because two patients received dexamethasone or placebo in a randomized controlled trial; #denotes less than 1 mg/kg of prednisone or equivalent
