# Supplementary material for: Risks of ventilator-associated pneumonia and invasive pulmonary aspergillosis in patients with viral acute respiratory distress syndrome related or not to Coronavirus 19 disease
Source: Crit Care. 2020 Dec 18;24:699. doi: 10.1186/s13054-020-03417-0 (PMC7747772; doi:10.1186/s13054-020-03417-0)
Supplement: Supplementary file 7 — Additional file 7. Table S6. Multivariable logistic regression of factors associated with invasive pulmonary aspergillosis (Influenza-Associated Pulmonary Aspergillosis case definition) in patients with acute respiratory distress syndrome related to Coronavirus disease 19 (C-ARDS) or other viruses (NC-ARDS). [file 13054_2020_3417_MOESM7_ESM.docx]

**Table S6. Multivariable logistic regression of factors associated with invasive pulmonary aspergillosis (Influenza Associated Pulmonary Aspergillosis case definition) in patients with acute respiratory distress syndrome related to Coronavirus disease 19 (C-ARDS) or other viruses (NC-ARDS).**

|  | **Missing**  **values, n (%)** | **Odd ratio (95% confidence interval), p value**  **by logistic regression** | |
| --- | --- | --- | --- |
|  |  | **Univariate** | **Multivariable** |
| Age | 0 | 1.04 (1.0-1.1), p=0.052 | I/NR |
| Immunodepression | 0 | 4.4 (1.7-10.7), p=0.001 | 3.6 (1.4-9.1), p=0.01 |
| Influenza | 0 | 2.9 (1.2-7.0), p=0.02 | 2.5 (0.88-6.4), p=0.052 |
| COVID-19 | 0 | 0.32 (0.13-0.82), p=0.02 | I/NR |

I/NR=included, but not retained by the final model. Abbreviations: COVID 19 coronavirus disease 2019. Of the significant univariate factors, only the most clinically relevant were entered into the regression model in order to minimize the effect of collinearity, as follows: Immunodepression was selected among Immunodepression, Lymphocyte count, Mc Cabe’s classification, and Charlson Comorbidity Index
